# COVID-19 Community Transmission and Super Spreaders in Rural Villages from Manabi Province in the Coastal Region of Ecuador Assessed by Massive Testing of Community-Dwelling Population

**DOI:** 10.4269/ajtmh.21-0582

**Published:** 2021-11-17

**Authors:** Maria Belén Rodriguez-Paredes, Paolo Alexander Vallejo-Janeta, Diana Morales-Jadan, Byron Freire-Paspuel, Esteban Ortiz-Prado, Aquiles R. Henriquez-Trujillo, Ismar A. Rivera-Olivero, Tatiana Jaramillo, Tannya Lozada, Miguel Angel Garcia-Bereguiain, on behalf of the UDLA COVID-19 Team

**Affiliations:** ^1^ One Health Research Group, Universidad de Las Américas, Quito, Ecuador;; ^2^ “UDLA-COVID-19 Team,” Universidad de Las Américas, Quito, Ecuador;; ^3^ Dirección General de Investigación, Universidad de Las Américas, Quito, Ecuador

## Abstract

Neglected rural communities in Latin America are highly vulnerable to COVID-19 due to a poor health infrastructure and limited access to severe acute respiratory syndrome coronavirus 2 (SARS-CoV-2) diagnosis. Manabí is a province of the Coastal Region of Ecuador characterized by a high prevalence of rural population living under poverty conditions. In the current study, we present the retrospective analysis of the results of a massive SARS-CoV-2 testing operation in nonhospitalized populations from Manabí carried out from August to September 2020. A total of 4,003 people from 15 cantons were tested for SARS-CoV-2 by reverse-transcriptase quantitative polymerase chain reaction, resulting in an overall infection rate of 16.13% for SARS-CoV-2, with several communities > 30%. Moreover, 29 SARS-CoV-2 super-spreader community-dwelling individuals with viral loads above 10^8^ copies/mL were found. These results support that uncontrolled COVID-19 community transmission was happening in Manabí during the first semester of COVID-19 pandemic. This report endorses the utility of massive SARS-CoV-2 testing among asymptomatic population for control and surveillance of COVID-19.

## INTRODUCTION

Coronaviruses (CoVs) are positive strand RNA viruses contained within a viral envelope with a crown-like morphology. They belong to the Nidovirus superfamily and are the largest known group of RNA viruses. Coronaviruses are the cause of many diseases in wild animals as well as domestic animals. In humans, the most prevalent coronavirus infections cause the common cold; however, severe acute respiratory syndrome (SARS)-associated coronaviruses have shown potential for severe, noteworthy diseases.^1^ On January 30, 2020, the WHO declared a “public health emergency of international concern” due to the outbreak of the novel coronavirus SARS-coronavirus 2 (CoV-2) to anticipate a coordinated international response.^2^ The SARS-CoV-2 pandemic spread from China to almost every country within months. In Latin America, the first outbreak appeared 4 weeks after Western Europe and 2 weeks after the United States and Canada, reaching marginalized regions with noticeable poverty. Because of the weak health infrastructure, understaffing, lack of biosafety equipment, and distrust in public governance, this region was greatly affected by the pandemic.^3^ During the first year of the COVID-19 pandemic, more than 51 million COVID-19 cases and more than 1 million deaths were reported in the Americas.^4^ More than 500,000 cases and 32,000 deaths associated with COVID-19 were reported since the first case in February 2020 through September 2021 in Ecuador.^5^

From the early stages of the COVID-19 pandemic, the WHO made a wide variety of recommendations, such as using face masks to reduce the spread of aerosol particles containing the virus, social distancing, and isolation of confirmed cases to slow the spread of the disease. One of the key observations of the WHO is that a successful surveillance strategy to contain the spread of COVID-19 is always based on testing as much of the population as possible. However, the Ecuadorian population has limited access to SARS-CoV-2 testing. In the early stages of the pandemic, only the National Institute of Research in Public Health laboratories, located in the three main cities of Ecuador (Guayaquil, Quito, and Cuenca) performed SARS-CoV-2 detection using reverse-transcriptase quantitative polymerase chain reaction (RT-qPCR) within the public health system, which was translated in a poor testing ratio of 7.46 PCR tests per 10,000 people.^6^ Up to September 10, 2021, after more than 1 year into the COVID-19 pandemic, 1,786,863 SARS-CoV-2 RT-qPCR tests had been done for 17 million Ecuadorians, with a positivity rate of 28.2%, according to the Ecuadorian Ministry of Health (MoH).^5^ The WHO recommends that no more than 5% of the individuals tested for SARS-CoV-2 should be positive to consider a surveillance program to control the spread of the virus. This means that many regions in Ecuador have not been sufficiently tested to control COVID-19 spread.^7^

Manabí is a primarily rural Ecuadorian province with an area of 19,427 km^2^ and a total of 1,390,200 inhabitants, of whom 617,880 reside in the rural areas. Manabí is the fourth largest province and the third most populated, with 22 counties.^8^ This province had a gross value added of 5,829.023 million USD in 2019 (gross value added for Ecuador was 100,871.577 million USD), giving a gross value added per capita below 5,000 USD (according to the data from Central Bank of Ecuador). On April 16, 2016, a 7.8-magnitude earthquake hit Ecuador with its epicenter in Pedernales, Manabí. The earthquake affected 720,000 people, with a total of 660 deaths, 4,605 injuries, 40 people missing, more than 30,000 people displaced, and 9,750 damaged buildings. The infrastructure damage included 59 hospitals and healthcare facilities, which, due to structural damage, were rendered inoperative in most cases. The impacted cities became chaotic with displacement and rural communities highly affected. The effect of the earthquake became visible with damage to the sanitation infrastructure, disruption of healthcare services, and overwhelming social and environmental disturbances. To this date, the province has not fully recovered from the infrastructure damages left by the earthquake.^9^ The lack of medical infrastructure, paired with RT-qPCR sampling limited to symptomatic patients attending a health center in urban areas, makes the rural areas of Manabí especially vulnerable to the SARS-CoV-2 pandemic. According to the MoH, during the first half year of the COVID-19 pandemic up to September 12, 2020, a total of 20,598 SARS-CoV-2 RT-qPCR tests done. This means that 1.5% of the Manabí population was tested at that point despite the dramatic 44.9% positivity rate.^10^ Up to May 2021, this province only has a small-capacity SARS-CoV-2 diagnosis laboratory within the public health system, meaning this region is still highly vulnerable to SARS-CoV-2 outbreaks.

This study is a retrospective analysis of epidemiological data obtained after massive aid surveillance SARS-CoV-2 testing carried out in coordination with local community leaders, the MoH, and the regional government (Prefectura de Manabi) at rural communities on 15 cantons included in the province of Manabí. This study is a follow-up of a previous short report published in this journal,^11^ now including all the community-dwelling individuals tested from August to September 2020.

## METHODS

### Study design and setting.

**Figure 1.**
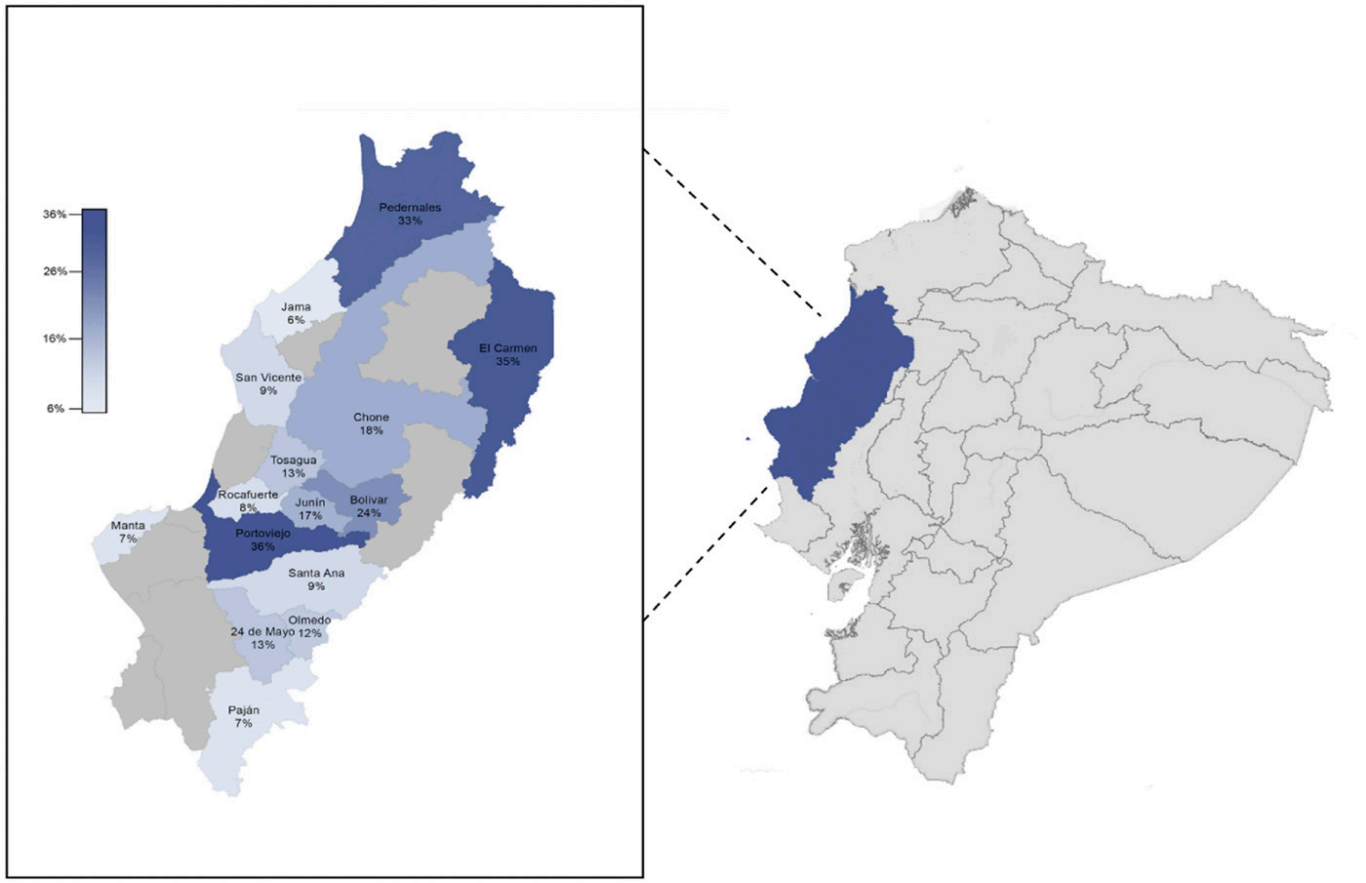
Location of Manabí Province in Ecuador. Cantons included in the massive testing during August–September 2020 and their severe acute respiratory syndrome coronavirus 2 infection rates. This figure appears in color at www.ajtmh.org.

A total of 4,003 individuals enrolled the surveillance. All samples were taken from community-dwelling, mostly asymptomatic individuals at the communities visited since August 3, 2020 to September 14, 2020, in 15 of 24 cantons of Manabí Province: Rocafuerte, Pajan, Santa Ana, Junín, Tosagua, Olmedo, Portoviejo, Manta, San Vicente, 24 de Mayo, Bolivar, Pedernales, Chone, El Carmen, and Jama (Figure 1).

Because the samples were not collected for a research study but as part of an aid surveillance intervention, the communities were selected by convenience following the recommendations from the local organizations and the provincial government of Manabí that helped us. Rural communities with high levels of poverty and reported cases of COVID-19 were included in the study. Within the community, convenience sampling was carried out where only one family member per household was included on the testing.

### Sample collection, RNA extraction, and RT-qPCR for SARS-CoV-2 diagnosis using the CDC protocol.

Nasopharyngeal swabs were collected on 0.5-mL Tris-EDTA (TE) pH 8 buffer for SARS-CoV-2 diagnosis by RT-qPCR following an adapted version of the CDC protocol by using PureLink Viral RNA/DNA Mini Kit (Invitrogen, Waltham, MA) as an alternate manual column-based RNA extraction method and CFX96 BioRad (Hercules, CA) instrument.^12^^–^^19^ Briefly, the CDC-designed RT-qPCR FDA EUA 2019-nCoV CDC kit (Integrated DNA Technologies, Coralville, IA) is based on N1 and N2 probes to detect SARS-CoV-2 and RNase P as an RNA extraction quality control.^18^^,^^19^ Also, negative controls (TE pH 8 buffer) were included as control for carryover contamination (one for each set of RNA extractions) to guarantee that only true positives were reported. For viral loads calculation, the 2019-nCoV N positive control (Integrated DNA Technologies) was used, provided at 200,000 genome equivalents per microliter, and a factor of 200 was applied to convert the viral loads to genome equivalents per milliliter and then converted to logarithmic scale.

### Statistical analysis.

For the statistical analysis of data, positivity rates were calculated for each canton, as well as for different age groups and sexes. To assess differences in the positivity rates, χ^2^ for comparison of proportions was applied. All statistical analysis was carried out using R software.

## RESULTS

**Figure 2.**
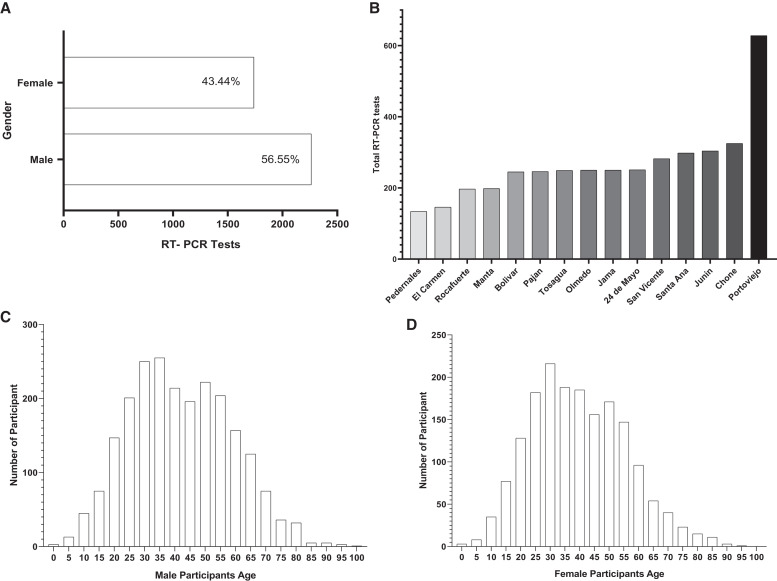
Description of the study population from Manabí included in the severe acute respiratory syndrome coronavirus 2 testing. (**A**) Distribution of individuals by sex. (**B**) Number of tests applied in each of the 15 cantons from Manabí Province. (**C** and **D**) Population distribution by age among male and female participants.

This study tested 4,003 people for SARS-CoV-2 using nasopharyngeal swabs and RT-qPCR, out of which 2,264 (56.55%) were male and 1,739 (43.44%) were female (Figure 2). The study was carried out in 15 cantons of Manabí province (Figures 1 and 2). The individuals tested at each canton were distributed as follows: 134 at Pedernales, 146 at El Carmen, 197 at Rocafuerte, 198 at Manta, 245 at Bolivar, 246 at Pajan, 249 at Tosagua, 250 at Olmedo, 250 at Jama, 251 at 24 de Mayo, 282 at San Vicente, 298 at Santa Ana, 304 at Junín, 325 at Chone, and 628 at Portoviejo. Most individuals recruited were between ages 30 and 40 years (males: mean= 37.63 ± 0.86; females: (mean = 38.42 ± 0.91), as detailed in Figure 2.

The SARS-CoV-2 infection rates were significantly different (*P* < 0.05) among the cantons included in the study (Figure 1): 33% for Pedernales, 35% for El Carmen, 8% for Rocafuerte, 7% for Manta, 24% for Bolivar, 7% for Pajan, 13% Tosagua, 12% Olmedo, 6% for Jama, 13% for 24 de Mayo, 9% for San Vicente, 9% for Santa Ana, 17% for Junín, 18% for Chone and 36% for Portoviejo.

**Figure 3.**
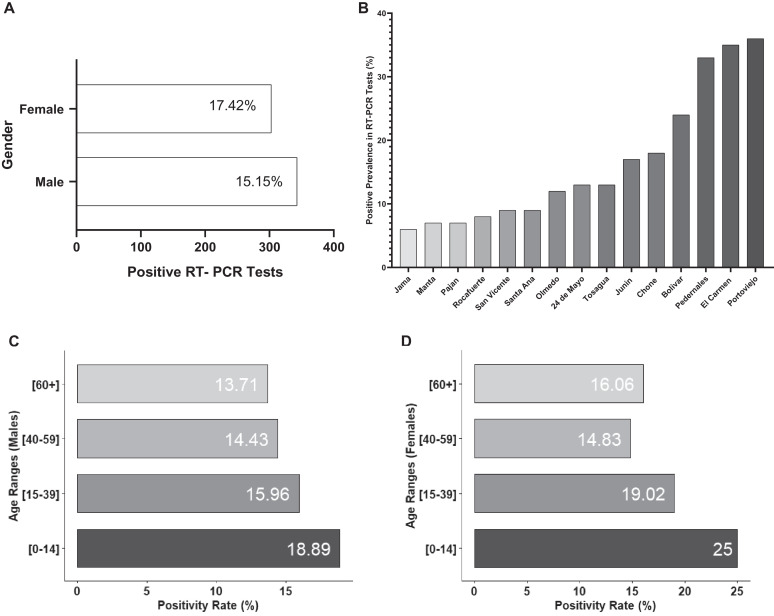
Positivity rates in the study population. (**A**) Total number of reverse-transcriptase quantitative polymerase chain reaction (RT-qPCR) tests per gender and their infection rate. (**B**) Positive rate of RT-qPCR tests per canton. (**C** and **D**) Positivity rate according to the age and sex of participants.

The overall SARS-CoV-2 infection rate was 16.13%, as 646 individuals tested positive for SARS-CoV-2 RT-qPCR. In Figure 3, the SARS-CoV-2 infection rate for male and females is shown, with values of 15.15% and 17.42%, respectively, although this differences were not significant (*P* > 0.05). Also, the highest percentage of SARS-CoV-2 positive cases was distributed among young males (mean = 38.59 ± 0.99) and young females (mean = 37.11 ± 0.96), as detailed on Figure 3.

**Figure 4.**
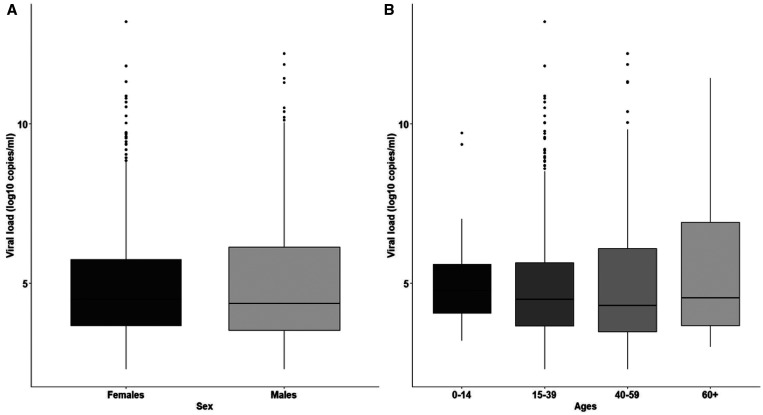
Severe acute respiratory syndrome coronavirus 2 viral load distribution among sex (**A**) and age (**B**) ranges (0–14: children; 15–39: young adults; 40–59; adults, 60+: elders).

Viral load distribution did not show any significant difference among sex (*P* > 0.05) or age groups (*P* > 0.05) (Figure 4). Thirty-nine individuals had SARS-CoV-2 viral loads values > 10^8^ copies/mL.

## DISCUSSION

More than 1 year since the first reported case of COVID-19 in Latin American, the epidemiological information available is scarce. Moreover, this information is mainly coming from government reports that are frequently incomplete for Ecuador.^6^^,^^7^^,^^11^^,^^20^^–^^23^ According to the few scientific reports available, SARS-CoV-2 community transmission was happening in Ecuador^11^^,^^21^^–^^23^ during the first half-year of the COVID-19 pandemic. Not enough testing capacity was available across the country, particularly among rural and indigenous communities.^6^^,^^7^^,^^20^ Furthermore, no information about the epidemiological situation of COVID-19 among community-dwelling asymptomatic individuals beyond the few studies carried out by universities is available.^7^^,^^11^^,^^21^^–^^23^ In this context, the present study is a follow-up of a previous short report publish in this journal.^11^ Now, with 15 cantons from Manabí Province included and more than 4,000 community-dwelling individuals tested, the SARS-CoV-2 infection rate of 16.13% clearly supports that uncontained SARS-CoV-2 community transmission was happening in Manabí Province during August–September 2020. Our study also highlights the limited SARS-CoV-2 diagnosis capacity installed in this province: the 4,003 samples collected for SARS-CoV-2 testing within a few weeks from August to September 2020 by our medical brigades represents 19.4% of the total 20,598 RT-qPCR test performed at Manabí Province up to September 12, 2020.^10^

Although the overall SARS-CoV-2 infection rate of 16.3% is high, the situation is particularly worrying in El Carmen, Portoviejo, and Pedernales cantons, with infection rates > 30%.

There was no clear association between high infection rates and proximity to urban locations, as the two main cities in Manabí Province are Manta and Portoviejo (both with populations greater than 150,000 people), located in cantons where the rural communities had either low or high infection rates, respectively. So far, a geographic trend was not found; high or low infection rates were evenly distributed across the province. All in all, our results confirm COVID-19 community transmission across the Coastal Region of Ecuador. Beyond the dramatic situation broadcasted by the media at Guayaquil during the initial COVID-19 outbreak in March 2020, COVID-19 community transmission has also been described for the coastal provinces of Santa Elena and Esmeraldas.^23^^,^^24^ Large population seroprevalence studies would be helpful to determine the dimension of COVID-19 spread on these communities from the Coastal Region of Ecuador.

Although any trend regarding SARS-CoV-2 viral loads associated with either sex or age was found at our study population, it is important to note that 39 individuals had viral loads ≥ 10^8^ viral copies/mL and could be considered SARS-CoV-2 super spreaders.^25^ Those community-dwelling individuals were either completely asymptomatic or reported some mild symptoms at the time of sample collection. This finding is particularly worrying considering that we did not observe a strong adherence to either mask use or social distancing in the communities surveyed.

The impact of COVID-19 pandemic was dramatic worldwide, but rural communities such as those described in this study for the Manabí Province in Ecuador were even more exposed and at risk of severe consequences from COVID-19 outbreaks. These communities have been traditionally neglected in terms of public health infrastructure. Moreover, the conditions imposed by climate and poverty in rural settings in the Ecuadorian coastal region make those communities prone to the spread of SARS-CoV-2,^23^^,^^26^^–^^30^ which indicates the necessity of the optimal implementation of control and prevention strategies for these neglected populations.

Although the main limitation of our work is that sampling was no randomized because samples were not collected for a research study but for an aid diagnosis program, we believe our results may nonetheless reflect the COVID-19 pandemic situation in Manabí during August–September 2020 for several reasons. First, we covered community spread in multiple cantons throughout the region. Second, several locations were visited within each canton. Third, only one person per household was included in the surveillance to exclude family-clustering bias in the infection rate calculation. Fourth, the sample size is > 4,000 individuals. Fifth, living conditions for the majority of the population in Manabí are similar to those in the communities visited.

In conclusion, we suggest than more resources should be allocated for COVID-19 pandemic containment in Manabí Province, from improving testing capacities to reinforcing hospital capacity to attend to COVID-19 patients under an scenario of community transmission such as that described in our study.
